# Triple platelet inhibition in intracranial thrombectomy with additional acute cervical stent angioplasty due to tandem lesion: a retrospective single-center analysis

**DOI:** 10.1186/s12883-024-03597-0

**Published:** 2024-03-18

**Authors:** Ali Khanafer, Hans Henkes, Philipp Bücke, Florian Hennersdorf, Hansjörg Bäzner, Michael Forsting, Philipp von Gottberg

**Affiliations:** 1 Neuroradiological Clinic, Neurozentrum, Klinikum Stuttgart, Stuttgart, Germany; 2 Institute for Diagnostic and Interventional Radiology and Neuroradiology, University Hospital Essen, Essen, Germany; 3 Department of Neurology, Inselspital, Bern University Hospital, University of Bern, Bern, Switzerland; 4 Department of Diagnostic and Interventional Neuroradiology, University of Tübingen, Tübingen, Germany; 5 Neurological Clinic, Neurozentrum, Klinikum Stuttgart, Stuttgart, Germany; 6 Klinik für Neuroradiologie, Klinikum Stuttgart, Kriegsbergstr. 60, 70174 Stuttgart, Germany

**Keywords:** Stroke, Carotid artery stenting, Endovascular thrombectomy, In-Stent restenosis, Anti-platelet therapy

## Abstract

**Background:**

Acute stroke treatment with intracranial thrombectomy and treatment of ipsilateral carotid artery stenosis/occlusion (“tandem lesion”, TL) in one session is considered safe. However, the risk of stent restenosis after TL treatment is high, and antiplatelet therapy (APT) preventing restenosis must be well balanced to avoid intracranial hemorrhage. We investigated the safety and 90-day outcome of patients receiving TL treatment under triple-APT, focused on stent-patency and possible disadvantageous comorbidities.

**Methods:**

Patients receiving TL treatment in the setting of acute stroke between 2013 and 2022 were analyzed regarding peri-/postprocedural safety and stent patency after 90 days. All patients received intravenous eptifibatide and acetylsalicylic acid and one of the three drugs prasugrel, clopidogrel, or ticagrelor. Duplex imaging was performed 24 h after treatment, at discharge and 90 days, and digital subtraction angiography was performed if restenosis was suspected.

**Results:**

176 patients were included. Periprocedural complications occurred in 2.3% of the patients at no periprocedural death, and in-hospital death in 13.6%. Discharge mRS score was maintained or improved at the 90-day follow-up in 86%, 4.54% had an in-stent restenosis requiring treatment at 90 days. No recorded comorbidity considered disadvantageous for stent patency showed statistical significance, the duration of the endovascular procedure had no significant effect on outcome.

**Conclusion:**

In our data, TL treatment with triple APT resulted in a low restenosis rate, low rates of sICH and a comparably high number of patients with favorable outcome. Aggressive APT in the initial phase may therefore have the potential to prevent recurrent stroke better than restrained platelet inhibition. Comorbidities did not influence stent patency.

**Supplementary Information:**

The online version contains supplementary material available at 10.1186/s12883-024-03597-0.

## Background

Since the publication of the trial data by MR CLEAN in 2014, endovascular mechanical thrombectomy of acute ischemic stroke has seen an upsurge in application, research, and dissemination that few methods in medicine experience.

However, new challenges have arisen from this constant development of endovascular stroke treatment. One common challenge is that associated with intracranial vessel occlusion treatment combined with an ipsilateral carotid artery stenosis or occlusion, a so called “tandem lesion” (TL). Many investigators have shown the feasibility and safety of treatment of both lesions in one session with a superiority in terms of thrombolysis-in-cerebral-infarction-(TICI-) scale [1] and clinical outcome [2] as well as a similar rate of adverse events compared with staged treatment [3].

However, the risk of stent re-occlusion is high in patients with a treated TL, and anti-platelet therapy (APT) must be carried out carefully since the brain infarction is prone to hemorrhage. Even under careful administration of anti-platelet medication, the risk of stent re-occlusion may be up to 57% in the first 24 h [4]. However, sustained stent patency after acute treatment of a TL is associated with improved clinical outcome [5–7].

Currently, data on the safety of APT in the course of endovascular TL treatment as well as applied drug strategies are inhomogeneous [8–10], making consent or recommendation of an anti-thrombotic medical regiment difficult. Furthermore, a recent meta-analysis did not find a significant difference in the use of no APT vs. single APT vs. dual APT as periprocedural platelet managements in TL treatment [11].

We therefore examined the stent patency and outcome in 176 consecutive patients at 24 h, discharge and 90 days following TL treatment with a homogeneous drug regimen of triple APT, which we assumed might be superior to the drug regimens described so far.

## Methods

### Study population

We analyzed data from all patients at our neurointerventional center who were treated for acute ischemic stroke with intracranial thrombectomy and ipsilateral carotid artery stenting (TL) between 2013 and 2022. To be included in this study, the causes of cervical internal carotid artery (ICA) lesions had to be atherosclerosis or dissection. TL Patients not treated with triple APT as described under “*Medication*” in the following section were excluded. TL Patients treated with carotis artery stenting (CAS) in the intradural segments of the ICA and vessel status < TICI2b at the end of intervention were excluded.

Patients’ medical records were analyzed for body mass index, diabetes, previous myocardial infarction, arterial hypertension, and smoking. Patients’ sex and age were recorded, and ICA stenosis grade was classified according to NASCET criteria [12]. We further recorded periprocedural data including duration of intervention, time from onset to recanalization, ASPECT score, TICI score, collateral score, use of percutaneous transluminal angioplasty (PTA) after stenting, number of stents deployed, and number of intracranial thrombectomy passes.

### Medication

Preceding ICA stent implantation, all patients received a single body-weight-adjusted intravenous bolus of Eptifibatide (135 mcg/kg body weight; Integrilin, GlaxoSmithKline). In addition, all patients received 500 mg acetylsalicylic acid (ASA; Aspirin i.v. 500 mg, Bayer Vital) intravenously and either 180 mg ticagrelor (Brilique, AstraZeneca), 40 mg prasugrel (Efient, Daiichi Sankyo), or 300 mg clopidogrel (Plavix, Sanofi-Aventis) via a nasogastric tube.

For medicinal treatment, 1 × 100 mg ASA and 2 × 90 mg ticagrelor or 1 × 10 mg prasugrel or 1 × 75 mg clopidogrel were administered from day 1 after treatment for 1 year. Thereafter 1 × 100 mg ASA was prescribed for life. Until 2015, the combination with clopidogrel was the standard, and we tested for sufficient inhibition within 12 h after stent implantation using a platelet function analyzer or multiplate analyzer (Roche Diagnostics, Mannheim, Germany) and VerifyNow® (Accriva, San Diego, CA, USA). Until 2015, clopidogrel malresponders were switched to ticagrelor. In cases of intolerance to ticagrelor (e.g. shortness of breath), we switched to prasugrel.

### Follow-up

All patients received duplex imaging for stent patency at 24 h (+/-6 h) following treatment, at discharge and 90 days (+/-10d) following treatment. If in-stent thrombosis and/or stenosis were suspected, digital subtraction angiography (DSA) was performed for confirmation.

The clinical endpoints within the 90-day follow-up were as follows:

- any periprocedural complication resulting in a major hemorrhagic event, major stroke, or death.
- mRS at discharge and at 90 days.
- any mortality related to the CAS procedure.
- any permanent clinical worsening associated with the CAS procedure.
- in-stent thrombosis or stenosis detected on duplex imaging, confirmed by DSA.
- any ipsilateral postprocedural stroke or major hemorrhagic event.
- any postprocedural complication resulting in a major hemorrhagic event, including symptomatic intracranial hemorrhage (sICH), major stroke, or death.

Major hemorrhagic events were defined as hemorrhages requiring an endovascular or surgical intervention or bleeding that lowered hemoglobin levels and required transfusion of red cell concentrate according to the definition from the International Society on Thrombosis and Hemostasis [13]. Severe stroke was defined as a permanent neurological deficit that resulted in a worsening of the modified Rankin Scale (mRS) score by at least one point. The decision to assess an ischemic event on the basis of an mRS deterioration was due to the retrospective nature of the data collection.

All surviving patients underwent neurological examination at discharge and at 90 days using the mRS. Implanted stents were also evaluated sonographically at discharge and at 90 days. If an in-stent stenosis was suspected, patients underwent DSA to exclude or confirm stenosis.

### Statistical analysis

Descriptive statistics were assessed as mean ± standard deviation for continuous variables and counts and percentages for categorical variables. The exact fisher-test was used to determine the endpoint *p*-values; the Mann–Whitney U Test was used for multivariate analyses. Also, multivariate analysis used a logistic regression model with stepwise selection. The threshold for statistical significance was set at 5% (0.05).

## Results

Between 2013 and 2022, 3636 intracranial thrombectomies were performed at our institution. In 266 cases, stent-assisted angioplasty of a cervical and/or intracranial vessel connected to the occluded intracranial segment was performed under triple APT in the anterior and posterior circulation and met the “equal or better TICI2b” criterion at the end of the intervention. Among these, 176 consecutive patients received cervical stent-angioplasty and ipsilateral intracranial thrombectomy under triple APT in the anterior circulation and were included in the analysis exclusively. The mean age was 69.1 years, the male gender was dominant at 63.1% (*n* = 111). Among the reported comorbidities, i.e., atrial fibrillation, arterial hypertension, coronary heart disease, diabetes mellitus, hyperlipidemia, and current tobacco smoking, arterial hypertension was the most common at 61.9% (Table 1).

**Table 1:** Comorbidities of included patients

		*n*	mRS 90 d		OR (95%-KI)	*p*-value*
			3–6	0–2		
pre mRS	0	101	45 (44.6%)	56 (55.4%)	-	
	1	39	23 (59.0%)	16 (41.0%)	0.56 (0.26–1.19)	0.136
	2	24	17 (70.8%)	7 (29.2%)	0.33 (0.12–0.89)	0.024
	3	12	12 (100%)	0 (0%)	-	< 0.001
AF	no	147	73 (49.7%)	74 (50.3%)		
	yes	29	24 (82.8%)	5 (17.2%)	0.21 (0.07–0.59)	0.001
diabetes	no	145	75 (51.7%)	70 (48.3%)		
	yes	31	22 (71.0%)	9 (29.0%)	0.44 (0.19–1.03)	0.072
cholesterol	no	149	86 (57.7%)	63 (42.3%)		
	yes	27	11 (40.7%)	16 (59.3%)	1.99 (0.86–4.61)	0.140
hypertension	no	67	41 (61.2%)	26 (38.8%)		
	yes	109	56 (51.4%)	53 (48.6%)	1.49 (0.80–2.78)	0.216
current tobacco smoking	no	138	77 (55.8%)	61 (44.2%)		
	yes	38	20 (52.6%)	18 (47.4%)	1.14 (0.55–2.34)	0.854
cardiac disease	no	132	62 (47.0%)	70 (53.0%)		
	yes	44	35 (79.5%)	9 (20.5%)	0.23 (0.10–0.53)	< 0.001
amount of comorbidities	0	21	13 (61.9%)	8 (38.1%)		
(AF-cardiac disease)	1	49	22 (44.9%)	27 (55.1%)		
	2	56	31 (55.4%)	25 (44.6%)		
	3	32	19 (59.4%)	13 (40.6%)		
	4	13	8 (61.5%)	5 (38.5%)		
	5	3	2 (66.7%)	1 (33.3%)		
	6	2	2 (100%)	0 (0%)		

* Exact Fisher-test

The side distribution of occluded ICAs was almost equal with 89 occlusions of the left ICA vs. 87 right-sided ICA. The cause of vessel stenosis or occlusion was atherosclerotic disease in 96.6% of cases and dissection in 3.4%. The degree of stenosis was 95% at mean (60–100%) following NASCET, and there were 30.7% complete occlusions.

The mean NIHSS score at presentation was 13.2. When comparing the mRS score at hospital admission with the “last seen well” score, the treated patients had suffered an average worsening of 3.64 points due to acute ischemic stroke leading to presentation at our department. 33% of patients received intravenous thrombolysis before MT. Thirteen patients received clopidogrel in the combination of antiplatelet agents, 139 patients received ticagrelor and 24 patients received prasugrel. Good response to APT was achieved in all patients after at least 12 h. The mean time from onset to puncture was 302.1 min, the mean time from puncture to recanalization TICI2b was 92.3 min. Please refer to Appendix for further details.

24 patients/13.7% died during the in-hospital phase, *n* = 7/4% of whom were directly connected to endovascular treatment (3 patients died of sICH following treatment, 4 patients died due to reperfusion syndrome, two of them with sICH). No patients showed restenosis or occlusion of the extracranial ICA at 24 h, and 8 patients (4.5%) showed a restenosis or occlusion 90 days after the intervention. There was no significant influence of one of any of the reported comorbidities on restenosis or re-occlusion, and there was no significant risk elevation in patients suffering from two or more of the reported comorbidities (Table 2).

**Table 2:** Comorbidities in relation to re-stenosis at 90 days

		*n*	Restenosis		OR (95%-KI)	*p*-value*
			no	yes		
AF	no	147	140 (95.2%)	7 (4.8%)		
	yes	29	28 (96.6%)	1 (3.4%)	0.71 (0.08–6.08)	1.000
diabetes	no	145	138 (95.2%)	7 (4.8%)		
	yes	31	30 (96.8%)	1 (3.2%)	0.66 (0.08–5.58)	1.000
cholesterol	no	149	141 (94.6%)	8 (5.4%)		
	yes	27	27 (100%)	0 (0%)		0.610
hypertension	no	67	65 (97.0%)	2 (3.0%)		
	yes	109	103 (94.5%)	6 (5.5%)	1.89 (0.37–9.74)	0.712
smoker	no	138	134 (97.1%)	4 (2.9%)		
	yes	38	34 (89.5%)	4 (10.5%)	3.94 (0.92–16.92)	0.067
cardiac disease	no	132	125 (94.7%)	7 (5.3%)		
	yes	44	43 (97.7%)	1 (2.3%)	0.42 (0.05–3.51)	0.681
Amount of comorbiditis	0	21	21 (100%)	0 (0%)		
(AF-cardiac disease)	1	49	47 (95.9%)	2 (4.1%)		
	2	56	52 (92.9%)	4 (7.1%)		
	3	32	30 (93.8%)	2 (6.2%)		
	4	13	13 (100%)	0 (0%)		
	5	3	3 (100%)	0 (0%)		
	6	2	2 (100%)	0 (0%)		
90 day mRS	0	24	23 (95.8%)	1 (4.2%)		
	1	33	30 (90.9%)	3 (9.1%)		
	2	22	21 (95.5%)	1 (4.5%)		
	3	23	20 (87.0%)	3 (13.0%)		
	4	32	32 (100%)	0 (0%)		
	5	11	11 (100%)	0 (0%)		
	6	31	31 (100%)	0 (0%)		

* Exact Fisher-Test

44.9% of the patients had a mRS score of 0–2 at the 90-day follow-up, 86.4% of the patients maintained (40.9%) or improved (45.5%) their discharge mRS score by follow up at 90 days. Improving patients had a mean reduction in mRS score of 1.7 points. Atrial fibrillation and coronary heart disease showed a significant connection with mRS 3–6 at 90 days (Table 1).

Furthermore, the duration of intervention and the number of comorbidities did not significantly influence the mRS score at 90 days. However, a high NIHSS at presentation was connected to a mRS score of 3–6 at 90 days (Table 3).

**Table 3:** Duration of intervention, NIHSS at presentation, and amount of comorbidities in relation to mRS at 90 days

	mRS90 d	*n*	Mv	Sd	Median	Min-Max	*p*-value* OR (95%-KI)
Puncture time to recanalisation in min	3–6	97	97.3	59.2	79.0	27.0-285.0	0.227
	0–2	79	86.8	54.4	68.0	32.0-311.0	1.00 (0.99-1.00)
NIHSS (Score)	3–6	94	15.6	6.5	17.0	0.0–27.0	< 0.001
	0–2	77	10.2	6.5	10.0	0.0–25.0	0.89 (0.86–0.93)
Amount of comorbidities	3–6	97	2.0	1.4	2.0	0.0–6.0	0.285
	0–2	79	1.8	1.1	2.0	0.0–5.0	0.87 (0.68–1.10)

* Mann-Whitney-U-Test

In the logistic regression (stepwise selection) as multivariate analysis, again only a high NIHSS at presentation showed a significant influence of the mRS at 90 days (Table 4).

**Table 4:** mRS 90 d - multivariate analysis (logistic regression); *n* = 171. In the full model, the mentioned characteristics from Tables 1 and 3 are taken into account. In the stepwise selection, the characteristics for which the *p*-value is below a specified level are selected from all characteristics using a stepwise procedure. This level was set at *p* < 0.05. To simplify matters, in the multivariate analysis, group 2 and 3 in pre mRS have been combined

	full model	stepwise selection (*p* < 0.05)
pre mRS 1 vs. 0	0.84 (0.31 ; 2.28), *p* = 0.729	0.76 (0.29 ; 1.97), *p* = 0.566
pre mRS 2–3 vs. 0	0.35 (0.12 ; 1.04), *p* = 0.059	0.30 (0.11 ; 0.86), *p* = 0.024
AF	0.21 (0.05 ; 0.84), *p* = 0.027	0.22 (0.07 ; 0.68), *p* = 0.009
diabetes	0.45 (0.15 ; 1.40), *p* = 0.169	-
cholesterol	3.22 (1.03 ; 10.11), *p* = 0.045	3.71 (1.23 ; 11.22), *p* = 0.020
hypertension	1.70 (0.76 ; 3.84), *p* = 0.199	-
Smoker	1.84 (0.64 ; 5.29), *p* = 0.257	-
cardiac disease	0.29 (0.12 ; 0.71), *p* = 0.007	0.35 (0.15 ; 0.85), *p* = 0.021
tPA	1.92 (0.87 ; 4.25), *p* = 0.106	-
Puncture time to recan. (min)	0.99 (0.99 ; 1.00), *p* = 0.125	-
NIHSS	0.87 (0.82 ; 0.93), *p* < 0.001	0.88 (0.83 ; 0.93), *p* < 0.001

## Discussion

This study focused on the outcomes for patients undergoing acute TL treatment under triple APT and analysis of the stent patency and outcome 24 h and 90 days after the procedure.

Extracranial stent patency at 24 h correlates with a better clinical outcome, and intravenous thrombolytic therapy accompanying thrombectomy was found to have a positive influence on stent patency. Recent studies advocate an aggressive APT in the initial phase to keep the stent open [7, 14, 15].

Among the patients included in this study, 33% (*n* = 58) received intravenous thrombolysis. However, stent patency at 24 h was 100% in our study, and there was no significant influence of thrombolysis on the patency of the stent for the extracranial carotid artery (Appendix).

The reported rates of restenosis or non-patency of the ICA after TL treatment at 24 h was reported to be 20.9% [16] in a recent large-volume multicenter analysis covering 225 patients; 22% [17] in a recent single-center analysis covering 99 patients with TL treatment through CAS; 55.4% in a single-center retrospective study covering 83 comparable patients [7]; and 56.9% in an analysis of two multicenter international observational registries covering 65 patients with comparable TL treatment [4] (Table 5).

**Table 5:** Comparison of preceding major case studies

Author (year)	*N*	ICA re-occlusion at 24 h	sICH	mRS 0–2 at 90d	APT
Allard et al. (2023) [14]	225	20.9%	n.a.*	54.7%	COXI and P2Y12 or GP-IIb/IIIa-I
Renú et al. (2020) [15]	99	22%	4%	66.7%	COXI and P2Y12
Bricout et al. (2018) [6]	83	55.4%	12.1%	41%	COXI
Marnat et al. (2020) [3]	65	56.9%	10.8%	54%	n.a.
Data in this study	176	0%	2.3%	45%	COXI and P2Y12 and GP-IIb/IIIa-I

Abbreviations: Cyclooxygenase Inhibitor - COXI; Glycoprotein IIb/IIIa inhibitor - GP-IIb/IIIa-I; P2Y12-antagnosit P2Y12. * Authors do not differentiate symptomatic and any intracerebral hematoma

With the method of APT unknown in Marnat et al., there are no comparable data on triple APT for TL treatment in literature and among the studies presented. In comparison, however, it is noticeable that there is already a clear difference in the proportion of ICA re-occlusion after 24 h between the studies with single platelet inhibition (Bricout et al.) and double platelet inhibition (Allard et al., Renú et al.) - to the disadvantage of single platelet inhibition.

This is only an imprecise comparison of the data based on the published results, which is still far from being reliable due to the differences in the centers, the patient population and treatment strategies. However, this trend also continues in our data, which show the lowest rate of 0% ICA re-occlusion after 24 h with the second largest number of cases.

The rate of extracranial ICA restenosis after 90 days in this study was 4.5% (*n* = 8). In this small case number, no influencing factor of significance could be identified. The number of patients with a mRS score of 0–2 at day 90 was within the range of published data, albeit in the lower section. The comparably low rate may be caused by the fact that patients included in this study had a long time from onset/last seen well to puncture (mean 302 min, range 66-3014 min).

Despite the fact that patients in our study had the most aggressive APT, the rate of sICH was the lowest within the mentioned studies.

It is particularly striking that when comparing the published results, there does not appear to be a trend towards higher bleeding rates with more aggressive platelet inhibition. Among the comparable data, Bricout et al. report a rate of sICH of 12.1% in 83 patients with single antiplatelet therapy vs. 13% in *n* = 225 total parenchymal hematomas (symptomatic and asymptomatic) in Allard et al. with double APT, 4% in *n* = 99 in Renú et al. with double APT and 2.3% in *n* = 176 in our data with triple APT.

A bias due to a possibly higher rate of sICH in other centres, for example due to a different type of focus on blood pressure monitoring, must not be assumed here. All of the data provided are data from neurovascular centres with a high and comparable level of experience and expertise. This raises the question of whether the occurrence of sICH may not necessarily be linked to the aggressiveness of platelet inhibition.

This would mean a rethink in the APT approach in emergency-based ICA stent implantation, but at the present time and with the data from this study, it is still far from evidence and requires additional or initial research.

Also, in contrast to a hyperperfusion syndrome as the cause for sICH, requiring patency of the formerly occluded or stenotic ipsilateral ICA, sICH following TL treatment may be more frequent in patients with stent-/ICA-reocclusion [6]. This assumption of a connection would be confirmed by the data presented here and the comparable studies.

Yet, data on factors influencing the development of sICH following TL treatment are too few, and also, in this study, no factor of significant influence could be identified. Still, in concordance with previously published data, the mortality of patients suffering from sICH is high at 50% in this study.

Furthermore, in accordance with previous reports [14], the correlation between higher NIHSS and a 90-day mRS score of 3–6 was found to be significant in our data. An inverse relationship was described for thrombectomy by Alexandre et al. They showed that patients with a low NIHSS also benefit from thrombectomy [18]. This finding is consistent with the authors’ opinion, which is why patients with low NIHSS were not discriminated for complex TL treatment in this study either.

A direct proportionality between time of intervention, number of recanalization attempts, and futile recanalization attempts on a negative, unfavorable outcome is well described for thrombectomy [19]. This however accounts for non-tandem, solitary lesions in the anterior circulation. Therefore, a question we wanted to investigate with our data was whether in the often-complex treatment of tandem lesions, supposedly originating from a chronic ICA stenosis, there is a point in intervention after which the chance of reversing stroke is overweighed by the risk of harming the patient, i.e., a time to stop.

However, regarding the 90-day mRS scores, there was no significant influence of the time from groin puncture to recanalization.

At a mean time of 92 min from groin puncture to recanalization TICI2b, the mean interventional treatment time was longer than treating an intracranial vessel occlusion alone, and nearly twice as long as in a 2016 meta-analysis on thrombectomy covering 1287 patients [20]. In this meta-analysis, a longer time to reperfusion was found to increase the risk of a worse outcome, congruent with other more recent studies on thrombectomy in purely intracranial vessel occlusion [15, 21, 22].

However, even in the group of patients undergoing interventional treatment with puncture-to-recanalization times beyond 120 min in our study (*n* = 44; 25%), only 3 patients/6.8% (1.7% in total) did not maintain or improve their discharge mRS at 90 days, but fell by one score point each.

The rate of in-hospital death in this subgroup was higher with *n* = 7/15.9% vs. *n* = 17/12.88% in the group of patients having endovascular procedures shorter than 120 min. However, all 7 patients with in-hospital death in the group with time-to-reperfusion beyond 120 min arrived at the hospital with a higher NIHSS (mean 14 [9–24] vs. 13 [14–27]) and had a longer time of onset to puncture (mean 366.7 min [196–622 min] vs. mean 302.16 min [66–871 min]). The latter two factors, known to have a negative effect on outcome, may be an explanation for the slightly higher in-hospital mortality rate in the group with the longer time from puncture to recanalization. Nevertheless, the cause of death was connected to the infarction in only 3 of the 7 patients in this study.

However, a lack of association between the timing of puncture and recanalization in TL could be explained by the fact that most TL patients probably had chronic moderate- to high-grade stenosis of the extracranial portion of the carotid artery that preceded the occlusion leading to clinical presentation. In such a setting, the brain may have already developed collateralization to cope with the chronically reduced blood flow, resulting in less severe cut of blood supply upon vessel occlusion. This consideration stems from the finding in the literature that in pure intracranial thrombectomy without CAS, the duration of the intervention until recanalization appears to have a negative influence on the outcome - which is at least described by the occurrence of intracerebral hemorrhage [23].

The number of patients with in-hospital death who had more than 3 attempts of intracranial thrombectomy is however high at 27.3% (6/22). 36.4%/*n* = 8 patients did not show improved mRS scored at discharge in this subgroup of patients, while only 27.3%/6 patients improved. This finding is concordant with the results of a major stroke registry analysis describing the odds for a favorable outcome of mechanical thrombectomy as the highest for patients with a maximum of 3 intracranial retrieval attempts [19].

One limitation of this study is the lack of an in-house comparison group with dual platelet inhibition - as is also the case with other studies with larger inclusion numbers, such as the studies cited in Table 5. A two-arm randomized controlled trial with double vs. triple platelet inhibition does not yet exist in the literature, which is why we have compared our study on triple APT with existing studies with single or double APT. The strength of this study is therefore, in addition to the novel principle of triple APT, the longer-term follow-up after TL treatment.

Many centers refrain from administering APT medication via a nasogastric tube due to the risk of complications such as aspiration or bleeding. Some centers are therefore shifting completely to intravenous administration of APT drugs at the time of stent implantation. We consider this to be completely justifiable, but the relevant experience and the appropriate drugs for pure intravenous dual platelet inhibition may be reserved for large neurovascular centers.

To minimize the risk of bleeding, in our practice the nasogastric tube is inserted immediately after intubation and before the administration of APT. The decision for or against a tube is made on the basis of the previous sectional imaging. According to our evaluation, the proportion of procedures in the course of which the indication for tube placement is first made is very low. Also, we can report no relevant rates of complications due to the nasogastric tube. However, due to different experiences in other departments, it may be that some departments would not wish to perform this practice. This can be seen as an imitation of this study.

Another possible limitation of this study is the fact that a potential anticoagulant status of the patients was not investigated. However, the cases described were ultimately acute patients treated under emergency conditions. In our analysis, therefore, reliable data on patients’ medication adherence prior to the stroke event were not available in all cases, and it is also difficult to assess patients’ medication adherence in the emergency situation.

Ultimately, we therefore analyzed the patients in this study regardless of their anticoagulation status, which may have biased the results to a certain extend.

## Conclusion

In TL treatment with triple APT, the rate of patent vessels in this data was higher compared to published data for dual or single APT at a comparable rate of favorable mRS at 90 days, but at a lower rate of sICH. Also, the restenosis rate after 90 days was low with triple APT. This suggests that an initially aggressive APT may be crucial for stent patency and that the rate of adverse events may be lower than expected. Triple APT could therefore be a so-far unused benefit for patients with tandem lesions and should be investigated further in a larger RCT.In addition, there is no relationship between the duration of the procedure and outcome in our data, suggesting that even if the procedure is laborious and difficult, revascularization should be pursued, as it can be a worthwhile endeavor.

### Electronic supplementary material

Below is the link to the electronic supplementary material.

Supplementary Material 1

## Data Availability

All data generated or analyzed during this study are included in this published article (and its supplementary information files).

## Declarations

### Concerning publishing

This manuscript has not been published elsewhere and is not under consideration by another journal. Data and results described in this manuscript have not been presented elsewhere.

## Abbreviations

APT: Anti platelet therapy
ASA: Acetyl salicylic acid
CAS: Carotid artery stenting
DSA: Digital subtraction angiography
ICA: Internal carotid artery
mRS: Modified ranking scale
PTA: Percutaneous transluminal angioplasty
sICH: Symptomatic intracranial hematoma
TICI: Thrombolysis in cerebral infarction
TL: Tandem lesion
