# Supplementary material for: Triple platelet inhibition in intracranial thrombectomy with additional acute cervical stent angioplasty due to tandem lesion: a retrospective single-center analysis
Source: BMC Neurol. 2024 Mar 18;24:99. doi: 10.1186/s12883-024-03597-0 (PMC10946095; doi:10.1186/s12883-024-03597-0)
Supplement: Supplementary file 1 — Supplementary Material 1 [file 12883_2024_3597_MOESM1_ESM.pdf]

**Sex**<br>**1: female**<br>**2: male**	**Age**	**Intervention Date**	**NIHSS (Score)**	**pre onset mRS** 1 to 6	**post onset mRS** 1 to 6	**AF**<br>**1: yes**<br>**2: no**
2	56	22.12.2013	14	0	4	2
2	69	23.12.2013	10	0	5	2
2	81	06.09.2014	8	0	4	2
1	51	22.11.2014	12	0	4	2
1	47	28.11.2014	5	0	4	2
1	51	23.12.2014	23	0	5	2
2	72	19.04.2015	18	0	5	2
2	71	20.04.2015	8	0	4	2
2	61	06.05.2015	26	0	5	2
2	50	11.05.2015	11	0	4	1
1	57	16.05.2015	2	0	5	2
2	73	14.06.2015	12	3	4	1
2	76	17.06.2015	20	0	4	2
2	71	14.07.2015	13	3	3	2
2	51	15.08.2015	6	0	4	2
2	83	10.09.2015	7	0	4	2
2	63	22.09.2015	1	0	3	2
1	85	01.11.2015	22	0	5	2
1	59	15.11.2015	2	0	2	2
2	75	16.12.2015	11	0	4	2
2	55	27.12.2015	n.a.	0	5	2
2	57	24.01.2016	6	0	4	2
1	54	18.04.2016	23	0	5	2
1	78	17.05.2016	10	0	4	2
1	53	06.07.2016	14	0	4	2
2	45	26.11.2016	1	0	1	2
2	54	27.11.2016	1	0	1	2
2	54	10.02.2017	14	2	5	2
1	68	27.02.2017	15	0	5	2
1	76	12.03.2017	7	0	4	2
1	78	28.03.2017	n.a.	1	5	2
2	57	29.03.2017	4	0	3	2
2	53	30.03.2017	12	1	4	2
2	49	27.04.2017	11	2	5	2
2	77	16.05.2017	11	0	5	2

1	90	20.05.2017	21	0	5	1
2	71	29.05.2017	21	0	5	2
2	79	08.03.2012	3	0	3	2
1	72	06.07.2017	18	0	5	2
2	49	05.07.2017	0	0	0	2
2	25	20.07.2017	2	1	2	2
1	57	23.07.2017	7	0	4	2
1	75	13.08.2017	8	2	3	2
1	79	23.08.2017	10	1	4	2
2	63	18.09.2017	21	2	5	2
2	77	29.09.2017	4	0	1	1
1	64	03.10.2017	17	0	4	2
2	81	16.11.2017	14	2	5	2
2	64	18.11.2017	18	0	5	2
2	77	22.12.2017	18	2	5	1
2	67	05.01.2018	21	0	5	2
1	59	06.01.2018	24	0	5	2
2	64	08.01.2018	8	1	4	2
2	57	09.02.2018	12	2	4	2
1	54	12.02.2018	15	0	4	2
2	73	03.03.2018	17	0	5	2
2	68	22.04.2018	16	1	4	2
2	79	04.05.2018	0	0	1	2
2	77	07.05.2018	15	0	5	2
1	78	24.06.2018	5	0	5	2
2	62	11.07.2018	16	0	4	2
1	77	27.07.2018	n.a.	0	5	2
2	57	19.08.2018	8	1	5	2
2	57	23.08.2018	2	0	2	2
1	55	09.11.2018	9	0	5	2
2	74	22.11.2018	17	0	4	2
1	75	07.12.2018	10	2	4	2
2	62	10.02.2019	1	0	3	2
2	75	02.03.2019	1	1	3	2
1	89	29.04.2019	25	1	5	2
2	82	30.04.2019	17	2	5	2
2	88	16.06.2019	3	0	4	1
2	74	24.08.2019	6	1	5	2
1	67	22.09.2019	22	1	5	2
1	63	29.10.2019	7	1	5	2
1	80	06.11.2019	2	1	3	1
1	56	01.12.2019	6	0	4	2
2	63	09.12.2019	8	1	4	2
2	48	20.12.2019	6	0	4	2
2	66	04.02.2020	10	0	4	2
2	70	11.06.2020	16	0	5	2

2	83	14.09.2020	4	2	4	2
1	80	09.10.2020	7	2	2	2
1	59	12.10.2020	7	0	5	2
2	58	21.10.2020	13	1	5	2
2	82	19.11.2020	19	1	4	2
1	70	30.11.2020	15	1	5	1
1	59	14.02.2021	15	0	4	2
2	78	23.02.2021	3	0	3	2
1	87	15.03.2021	16	1	5	1
2	67	28.03.2014	13	0	4	2
2	60	07.03.2015	21	0	5	1
2	76	26.03.2015	n.a.	0	5	2
1	71	27.03.2015	1	0	1	2
2	71	04.04.2015	17	0	5	2
1	78	07.05.2015	5	1	4	2
2	84	02.06.2015	20	0	5	2
1	70	24.06.2015	4	1	2	2
1	59	01.08.2015	16	0	5	2
1	86	01.09.2015	22	1	5	1
2	53	24.12.2015	13	0	4	2
1	88	01.06.2016	12	0	5	2
2	70	13.07.2016	8	0	4	2
2	79	29.11.2016	18	0	5	2
2	73	02.12.2016	25	0	5	2
2	74	02.12.2016	24	2	5	2
2	79	11.02.2017	18	0	5	2
1	79	16.02.2017	3	1	4	1
1	83	16.02.2017	25	3	5	2
2	68	08.03.2017	15	0	5	2
2	59	18.03.2017	11	0	3	2
1	59	08.04.2017	18	1	5	2
2	85	13.06.2017	18	0	5	1
2	59	23.07.2017	26	0	5	2
1	86	31.07.2017	14	1	4	1
2	73	01.12.2017	14	0	5	2
2	62	06.12.2017	16	1	5	2
2	55	02.03.2018	27	1	5	1
1	75	08.03.2018	20	1	5	2
2	83	02.04.2018	17	2	5	2
2	80	29.06.2018	16	1	5	2
2	80	20.08.2020	20	2	5	2
2	68	14.09.2020	10	0	4	2
1	59	02.12.2020	18	1	5	2
2	70	09.10.2020	20	3	5	1
1	87	11.11.2020	9	2	4	1
1	83	29.11.2020	9	1	3	2

1	90	31.12.2020	0	3	5	1
2	72	05.01.2021	10	1	5	2
2	51	25.03.2021	20	0	5	2
2	49	29.03.2021	5	0	3	2
1	72	25.08.2018	4	1	4	2
1	76	03.09.2018	12	3	4	1
2	82	16.08.2018	6	1	4	1
1	81	18.09.2018	21	2	5	2
2	79	01.10.2018	11	0	5	1
1	78	05.11.2018	12	0	4	2
2	73	14.11.2018	13	3	4	2
2	94	03.12.2018	20	3	5	1
1	78	21.12.2018	3	2	5	1
2	67	22.12.2018	14	0	5	1
2	54	25.12.2018	16	0	5	2
2	77	31.12.2018	22	0	5	2
1	68	08.01.2019	20	2	5	2
2	65	16.01.2019	18	0	5	2
1	63	22.01.2020	10	0	5	2
1	77	10.02.2020	18	0	5	2
1	71	16.02.2020	13	0	4	2
1	75	23.02.2020	n.a.	2	5	2
1	65	16.03.2020	11	3	4	2
2	70	26.03.2020	14	0	5	2
2	79	02.06.2020	15	2	5	1
2	74	05.09.2016	15	0	4	2
1	72	14.08.2016	7	1	4	2
2	75	13.08.2015	18	0	5	2
2	50	05.06.2018	7	0	5	2
2	64	07.02.2019	19	1	5	2
1	84	13.06.2019	24	1	5	1
2	64	02.07.2019	22	2	5	2
1	89	06.07.2019	20	2	5	2
2	63	09.08.2019	27	3	5	2
2	77	12.08.2019	20	1	5	1
2	62	21.11.2019	18	0	5	2
2	60	24.04.2019	21	1	5	2
2	66	13.05.2019	20	0	5	2
2	76	26.06.2020	23	2	5	2
1	60	24.07.2020	8	0	4	2
2	66	15.08.2020	14	0	5	2
2	71	09.07.2018	17	1	5	2
1	89	01.11.2016	14	2	5	2
2	85	24.02.2017	21	3	5	1
2	64	18.07.2017	24	3	5	1
2	62	25.09.2017	2	0	5	2

2	65	31.05.2018	19	0	5	2
2	56	09.11.2015	14	0	5	2
2	61	06.02.2016	23	0	5	2

diabetes<br>1: yes<br>2: no	Cholesterol<br>1: yes<br>2: no	hypertension<br>1: yes<br>2: no	smoker<br>1: yes<br>2: no	cardiac disease e.g. myocardial infarction<br>1: yes<br>2: no	tPA or tenecteplase<br>1:tPA<br>3:no	intrcranial Occluded Vessel (ICA, M1, M2, ACA, Multi-territory)<br>0: no<br>ICA:1/ M1: 2 /M2:3/ACA:4 /<br>Multiterr
2	2	2	2	1	3	2
1	2	1	2	1	3	2
1	2	1	2	2	3	2
2	2	1	2	2	3	3
2	2	2	2	2	3	2
2	2	2	1	2	1	3
2	2	2	2	1	1	3
2	2	1	2	2	3	1
2	2	1	1	2	1	2
1	2	2	1	2	3	2
1	2	1	2	2	3	2
2	2	2	2	2	3	2
2	2	1	2	2	3	1
1	1	2	2	2	1	2
2	2	1	1	2	3	1
1	2	1	2	2	1	1
2	2	1	2	2	3	3
2	2	1	2	1	3	1
2	2	1	2	2	3	1
1	2	1	2	2	3	2
2	2	2	2	2	1	1
2	2	1	1	2	1	2
2	2	2	1	2	3	2
2	2	1	2	2	3	2
2	2	2	2	2	1	1
2	2	2	1	2	1	3
2	2	2	2	2	3	3
2	2	1	2	1	3	2
2	2	1	2	2	1	3
2	2	1	1	2	3	2
2	1	1	1	1	3	2
2	2	1	1	2	3	2
2	2	1	1	2	3	2
2	2	2	1	2	3	2
2	1	1	2	1	1	2

1	1	1	2	1	3	3
2	2	1	2	2	3	2
2	1	1	2	2	3	2
2	2	1	2	2	3	2
2	2	1	2	2	3	2
2	2	2	1	2	3	2
1	2	1	2	2	1	3
2	2	1	2	2	3	2
2	2	2	2	1	3	2
2	1	1	1	2	3	3
2	1	1	2	2	1	3
1	2	1	2	2	1	1
2	2	2	1	2	3	2
2	2	2	2	2	1	2
1	1	1	2	1	3	2
2	1	1	2	1	3	2
1	2	1	1	2	1	2
2	2	2	1	2	3	2
2	2	2	2	2	1	2
2	2	1	1	2	1	1
2	2	1	2	2	1	3
2	1	2	2	2	3	2
2	1	1	2	2	3	3
2	2	1	2	2	1	1
2	1	1	2	2	3	2
2	2	2	2	2	3	2
2	2	2	2	2	3	2
1	2	1	2	2	1	2
2	2	2	2	2	3	1
2	2	1	1	1	1	2
2	1	2	2	2	1	3
2	2	2	2	2	3	1
2	2	2	1	1	1	3
2	2	1	2	2	1	2
2	2	2	2	2	3	2
2	2	2	2	2	3	2
2	2	2	2	1	1	2
2	1	1	2	2	3	2
2	2	2	2	2	3	2
2	2	1	2	2	3	2
2	2	1	2	2	3	3
2	2	1	1	2	1	2
2	2	1	2	2	3	2
2	2	2	2	2	1	3
2	2	2	2	2	1	2
2	2	1	2	1	1	2

1	1	1	2	1	3	1
2	1	1	2	2	3	3
2	2	2	2	2	1	3
2	2	2	2	2	3	3
2	2	1	2	2	3	1
2	2	1	2	2	3	2
2	2	1	1	2	3	3
2	2	2	2	2	1	3
2	2	1	2	2	3	1
2	2	1	1	1	3	2
2	2	2	1	2	3	3
2	2	1	1	2	3	2
2	2	1	2	2	1	3
2	2	1	2	2	1	1
2	1	1	2	2	3	2
1	2	1	2	2	3	2
2	2	1	1	2	3	2
2	2	1	1	2	3	1
2	2	2	2	2	3	1
2	1	1	2	2	3	2
2	2	1	2	2	1	2
2	2	2	2	2	3	2
2	2	1	2	2	3	2
2	1	1	2	2	1	2
2	2	2	2	2	3	2
2	2	2	2	2	3	2
2	2	1	2	2	3	2
1	2	1	2	1	3	1
2	2	1	2	2	3	2+4
2	2	2	2	2	3	3
1	2	2	2	2	3	2
2	2	1	2	2	3	2
2	2	2	1	2	3	2
2	2	1	2	1	1	3
2	1	1	2	2	1	2
1	2	2	1	1	1	2
1	2	2	2	2	3	1
2	2	2	2	2	3	1
2	2	2	2	1	1	2
2	2	1	2	2	3	2
2	2	1	2	2	1	2
2	2	2	2	2	1	2
2	2	2	2	2	3	1
2	1	1	1	1	1	3
2	1	1	2	1	3	2
2	2	2	2	2	3	3

2	2	1	2	2	3	3
2	2	2	2	2	1	3
1	2	1	2	1	3	2
2	2	1	1	2	3	1
2	2	1	2	1	1	3
2	2	1	2	1	3	1
1	2	1	2	1	3	3
1	2	1	2	2	3	2
2	1	1	2	2	3	3
2	2	2	2	2	1	2
2	2	1	1	1	3	1
2	2	2	2	1	3	2
1	2	2	2	1	3	2
2	2	2	2	1	3	1
1	2	1	2	1	1	2
2	1	1	2	2	3	1
2	2	1	1	2	3	2
2	2	2	2	2	3	2
2	2	1	2	2	3	2
2	1	1	2	2	3	2
2	2	2	2	2	3	1
2	2	2	2	2	3	2
2	2	1	2	1	3	1
2	2	2	2	1	3	1
2	1	1	2	2	3	2
2	2	2	2	2	1	3
1	2	1	2	2	3	2
2	2	1	2	2	3	1
2	1	2	2	2	3	1
1	2	1	1	1	1	2
2	2	1	2	1	1	1
1	2	2	1	2	1	2
2	2	1	2	2	3	2
2	2	2	2	1	3	2
2	2	2	2	1	3	2
2	2	1	2	2	1	2
1	2	1	2	2	1	2
2	2	1	2	1	3	2
2	2	1	2	1	1	2
2	2	1	2	1	3	2
2	2	1	2	2	3	1
2	2	1	2	1	1	1
1	2	1	2	2	1	2
2	2	1	2	1	1	1
1	2	1	1	1	1	2+4
1	2	1	2	2	3	2

2	2	2	2	2	3	2
2	2	2	1	2	3	1
2	2	2	1	2	3	2

laterality (R/L) 1: right 2: left	cause of stenosis - 1= atherosclerosis, 2= dissection, other	degree of stenosis of ICA (ipsilateral) Nascet	ASPECT score	Time of onset to puncture in min	Puncture time to recanalisation in min	Time of onset to recanalisation in min
1	1	90	10	265	93	358
2	1	100	9	n.a.	78	n.a.
1	1	100	9	168	54	222
2	2	100	7	259	126	385
2	2	100	8	118	299	417
2	2	100	10	360	268	628
1	1	100	9	288	66	354
2	1	100	10	403	182	585
2	1	100	10	260	93	353
1	1	100	10	n.a.	124	n.a.
1	2	100	8	145	311	456
1	1	100	7	n.a.	97	n.a.
1	1	100	9	156	78	234
2	1	95	10	468	74	542
2	1	100	10	102	65	167
1	1	100	10	306	46	352
2	1	85	9	523	95	618
2	1	95	9	n.a.	41	n.a.
1	1	95	7	294	57	351
2	1	100	9	138	44	182
1	2	100	7	203	68	271
1	1	100	1	202	119	321
2	1	60	9	136	130	266
1	1	85	9	208	166	374
1	1	85	9	367	72	439
2	1	100	9	187	119	306
1	1	100	8	132	128	260
1	1	100	10	204	125	329
1	1	100	6	n.a.	97	n.a.
2	1	100	9	225	48	273
2	1	85	9	n.a.	146	n.a.
1	1	100	9	174	126	300
2	1	100	9	n.a.	120	n.a.
1	1	100	5	n.a.	173	n.a.
2	1	100	10	216	52	268

2	1	80	10	299	39	338
2	1	95	8	216	98	314
1	1	100	8	215	137	352
2	1	100	10	n.a.	109	n.a.
1	1	60	8	n.a.	75	n.a.
1	1	100	10	294	215	509
1	1	80	6	216	194	410
2	1	65	10	n.a.	96	n.a.
1	1	98	8	201	39	240
1	1	100	7	n.a.	89	n.a.
1	1	100	8	368	68	436
1	2	80	7	205	63	268
2	1	100	8	n.a.	94	n.a.
2	1	100	8	248	80	328
2	1	100	10	148	47	195
2	1	90	6	95	46	141
2	1	90	9	136	33	169
1	1	98	8	371	49	420
2	1	100	6	330	89	419
2	1	100	8	168	55	223
2	1	90	9	n.a.	53	n.a.
1	1	100	8	220	48	268
1	1	100	8	n.a.	93	n.a.
1	1	80	7	588	66	654
1	1	98	7	191	61	252
2	1	100	7	509	51	560
1	1	100	7	929	73	1002
1	1	98	10	268	54	322
1	1	100	9	174	64	238
2	1	100	10	182	58	240
2	1	100	9	204	32	236
1	1	75	10	1087	98	1185
1	1	100	10	186	43	229
1	1	98	10	205	66	271
1	1	75	9	265	51	316
1	1	75	8	262	79	341
2	2	80	10	218	43	261
1	1	90	9	277	41	318
2	1	100	5	n.a.	36	n.a.
1	1	90	8	268	54	322
2	1	100	9	n.a.	40	n.a.
1	1	90	9	575	62	637
1	1	100	7	382	139	521
1	2	100	10	n.a.	151	n.a.
2	1	100	8	233	51	284
1	1	98	9	248	66	314

1	1	100	8	n.a.	96	n.a.
1	1	100	8	n.a.	77	n.a.
2	1	98	9	342	78	420
2	1	98	8	376	43	419
2	2	100	10	n.a.	83	n.a.
2	1	100	8	187	83	270
1	1	100	6	n.a.	105	n.a.
2	1	100	9	3014	228	3242
1	1	80	10	n.a.	45	n.a.
1	1	60	10	198	63	261
2	1	95	10	n.a.	259	n.a.
1	1	100	7	116	254	370
1	1	70	5	162	130	292
1	1	100	9	467	56	523
1	1	90	7	n.a.	132	n.a.
2	1	70	10	307	44	351
2	1	100	10	197	70	267
2	1	100	8	n.a.	66	n.a.
2	2	70	9	240	175	415
1	1	100	6	171	75	246
2	1	99	8	398	57	455
2	1	100	9	n.a.	124	n.a.
1	1	100	8	179	170	349
2	1	100	8	226	52	278
1	1	100	10	622	171	793
1	1	100	7	n.a.	136	n.a.
2	1	80	8	n.a.	73	n.a.
2	1	100	6	n.a.	104	n.a.
1	2	100	9	192	255	447
1	1	100	8	n.a.	41	n.a.
2	1	100	8	378	80	458
1	1	100	9	116	54	170
2	1	100	9	n.a.	101	n.a.
1	1	100	9	230	90	320
2	1	100	8	175	120	295
2	1	100	10	380	136	516
2	1	100	8	n.a.	102	n.a.
1	1	100	7	n.a.	156	n.a.
1	1	100	7	169	80	249
2	1	100	7	66	79	145
1	1	85	10	270	37	307
2	1	80	8	200	83	283
1	1	100	7	n.a.	63	n.a.
1	1	100	10	340	50	390
1	1	90	9	223	175	398
2	1	100	10	196	127	323

1	1	98	10	377	60	437
1	1	100	8	267	40	307
1	1	100	6	n.a.	102	n.a.
2	1	95	9	119	66	185
2	1	100	10	229	121	350
1	1	100	10	185	68	253
1	1	70	10	392	70	462
1	1	100	10	460	79	539
2	1	100	10	100	37	137
2	1	98	9	199	38	237
1	1	100	9	249	38	287
1	1	100	10	380	60	440
2	1	100	9	265	52	317
2	1	100	9	273	61	334
2	1	100	8	170	58	228
2	1	99	10	n.a.	53	n.a.
2	1	100	9	194	131	325
1	2	100	9	140	176	316
2	1	100	7	n.a.	242	n.a.
2	1	98	9	153	46	199
2	1	85	7	n.a.	60	n.a.
2	1	100	10	247	27	274
1	1	100	4	365	140	505
1	1	100	4	n.a.	40	n.a.
2	1	100	8	356	48	404
2	1	100	10	265	102	367
1	1	100	9	n.a.	56	n.a.
2	1	90	10	117	49	166
1	1	90	10	n.a.	49	n.a.
2	1	100	8	327	43	370
2	1	100	9	236	49	285
2	1	100	10	n.a.	197	n.a.
1	1	75	10	n.a.	51	n.a.
2	1	60	9	n.a.	53	n.a.
1	1	95	8	n.a.	58	n.a.
2	1	100	9	1303	52	1355
2	1	100	5	n.a.	70	n.a.
2	1	100	9	199	194	393
2	1	100	6	871	76	947
1	1	95	10	243	50	293
2	1	100	10	n.a.	47	n.a.
2	1	100	9	n.a.	108	n.a.
1	1	100	8	n.a.	59	n.a.
2	1	100	8	n.a.	82	n.a.
2	1	80	9	n.a.	139	n.a.
2	1	100	10	271	70	341

1	1	100	10	357	91	448
1	1	90	8	n.a.	104	n.a.
2	1	75	10	232	285	517

TICI score end procedure	intracranial Technique (stent-retriever:1, aspiration:2, combined:3)	Number of attempts intracranial	Balloon angioplasty performed (Y:1/N:2)	stent type implanted	stenosis post treatment (Y:1/N:2)
2b	3	1	1	Wallstent	2
3	3	2	1	Wallstent	2
3	3	1	1	Wallstent	2
2b	3	1	1	p64, Wallstent	2
2b	3	4	1	Wallstent	2
3	3	1	1	Wallstent + p64	2
3	3	1	1	Wallstent	2
2b	3	7	1	Wallstent	2
3	3	1	1	Wallstent	2
2c	3	3	1	Wallstent	2
2b	3	2	1	Wallstent	2
2c	3	1	1	Wallstent	2
2c	3	2	1	Wallstent	2
2b	3	2	1	Wallstent	2
3	3	1	1	Wallstent	2
3	3	1	1	Wallstent	2
2b	3	2	1	Wallstent	2
3	3	1	1	Wallstent	2
3	3	1	1	Wallstent	2
3	3	1	1	Wallstent	2
3	3	1	1	Wallstent	2
2b	3	2	1	Wallstent	2
3	3	2	1	ent 7 x 40, Cguard Carotid	2
2b	3	4	1	Wallstent	2
3	3	2	1	Wallstent	2
3	3	2	1	Wallstent	2
3	3	3	1	Wallstent	2
2b	3	3	1	Wallstent	2
2c	3	1	1	Wallstent	2
2c	3	1	1	Wallstent	2
3	3	3	1	Wallstent	2
2b	3	4	1	Wallstent	2
2c	3	3	1	Wallstent	2
3	3	2	1	Wallstent	2
2c	3	1	1	Wallstent	2

3	3	2	1	Wallstent	2
3	3	3	1	Wallstent	2
3	3	2	1	Wallstent	2
3	3	2	1	Wallstent	2
3	3	2	1	Wallstent	2
2b	3	7	1	Wallstent	2
2b	3	5	1	Wallstent	2
2c	3	2	1	Wallstent	2
3	3	1	1	Wallstent	2
2b	3	1	1	Wallstent	2
2c	3	2	1	Wallstent	2
2b	3	2	1	Wallstent	2
2b	3	2	1	Wallstent	2
2c	3	2	1	Wallstent	2
3	3	1	1	Wallstent	2
3	3	1	1	Wallstent	2
2b	3	1	1	Wallstent	2
3	3	1	1	Wallstent	2
2b	3	1	1	Wallstent	2
2c	3	1	1	Wallstent	2
2b	3	2	1	Wallstent	2
3	3	2	1	Wallstent	2
3	3	2	1	Wallstent	2
3	3	2	1	Wallstent	2
3	3	1	1	Wallstent	2
2b	3	1	1	Wallstent	2
2b	3	1	1	Wallstent	2
3	3	1	1	Wallstent	2
2b	3	1	1	Wallstent	2
3	3	1	1	Wallstent	2
3	3	1	1	Wallstent	2
2b	3	3	1	Wallstent	2
2b	3	1	1	Wallstent	2
2b	3	1	1	Wallstent	2
3	2	1	1	Wallstent	2
2b	3	2	1	Wallstent	2
3	3	2	1	Wallstent	2
2b	3	1	1	Wallstent	2
2b	3	1	1	Wallstent	2
2b	3	1	1	Wallstent	2
3	3	2	1	Wallstent	2
2b	3	2	1	Wallstent	2
2b	3	4	1	Wallstent	2
3	3	1	1	Wallstent	2
2b	3	2	1	Wallstent	2
3	3	1	1	Wallstent	2

3	3	2	1	Wallstent	2
2c	3	2	1	Wallstent	2
3	3	1	1	Wallstent	2
2c	3	1	1	Wallstent	2
3	3	6	1	Wallstent	2
2b	3	3	1	Wallstent	2
2b	3	1	1	Wallstent	2
3	3	3	1	Wallstent	2
3	3	1	1	Wallstent	2
2c	3	2	1	Wallstent	2
2b	3	5	1	Wallstent	2
2b	3	8	1	Wallstent	2
2b	3	1	1	Wallstent	2
2b	3	2	1	Wallstent	2
2b	3	3	1	Wallstent	2
3	3	1	1	Wallstent	2
3	3	1	1	Wallstent	2
3	3	2	1	Wallstent	2
2b	3	7	1	Wallstent	2
3	3	2	1	Wallstent	2
3	3	1	1	Wallstent	2
3	3	1	1	Wallstent	2
3	3	3	1	Wallstent	2
3	3	1	1	Wallstent	2
3	3	4	1	Wallstent	2
2b	3	3	1	Wallstent	2
2b	3	1	1	Wallstent	2
3	3	2	1	Wallstent	2
2b	3	5	1	Wallstent	2
2b	2	2	1	Wallstent	2
2c	3	1	1	Wallstent	2
2c	3	1	1	Wallstent	2
2c	3	2	1	Wallstent	2
3	3	3	1	Wallstent	2
3	3	1	1	Wallstent	2
2B	3	3	1	Wallstent	2
2b	3	5	1	Wallstent	2
3	3	6	1	Wallstent	2
2b	3	2	1	Wallstent	2
2b	2	1	1	Wallstent	2
3	3	1	1	Wallstent	2
2C	3	2	1	Wallstent	2
3	3	2	1	Wallstent	2
3	3	2	1	Wallstent	2
2B	3	2	1	Wallstent	2
3	3	3	1	Wallstent	2

3	3	2	1	Wallstent	2
2C	3	2	1	Wallstent	2
3	3	2	1	Wallstent	2
3	2	1	1	Wallstent	2
3	3	3	1	Wallstent	2
2B	3	3	1	Wallstent	2
3	3	3	1	Wallstent	2
2B	3	1	1	Wallstent	2
3	3	1	1	Wallstent	2
3	3	1	1	Wallstent	2
3	3	1	1	Wallstent	2
3	3	2	1	Wallstent	2
2B	3	1	1	Wallstent	2
3	3	2	1	Wallstent	2
3	3	1	1	Wallstent	2
3	3	1	1	Wallstent	2
2b	3	4	1	Wallstent	2
3	3	3	1	Wallstent + Pipline	2
2B	3	6	1	Wallstent	2
2B	3	1	1	Wallstent	2
2B	3	1	1	Wallstent	2
3	2	2	1	Wallstent	2
2B	3	3	1	Wallstent	2
3	3	1	1	Wallstent	2
2C	3	1	1	Wallstent	2
2B	3	2	1	Wallstent	2
3	3	1	1	Wallstent	2
3	3	2	1	Wallstent	2
3	3	1	1	Wallstent	2
3	3	1	1	Wallstent	2
2B	3	2	1	Wallstent	2
2B	3	4	1	Wallstent	2
2B	3	1	1	Wallstent	2
2B	3	1	1	Wallstent	2
3	3	2	1	Wallstent	2
2B	3	2	1	Wallstent	2
2B	3	1	1	Wallstent	2
2B	3	1	1	Wallstent	2
3	3	1	1	Wallstent	2
3	3	1	1	Wallstent	2
2C	3	1	1	Wallstent	2
2B	3	4	1	Wallstent	2
3	2	1	1	Wallstent	2
2b	2	2	1	Wallstent	2
2B	3	6	1	Wallstent	2
2b	3	2	1	Wallstent	2

2b	3	2	1	Wallstent	2
2B	3	5	1	Wallstent	2
3	3	3	1	Wallstent	2

**Integrilin bolus Dose**	**Aspirin (dose)**	P2Y12 inhibitor (name and dose) 1= Brilique 180 mg 2=600 Plavix 3=60 Efient	Complications from procedure (Y:1/N:2)	sICH (Y:1/N:2)	Carotid Stent Restenosis 24 h (Y:1/N:2)	Discharge mRS (score)
14	500	1	0	2	2	5
10	500	1	0	2	2	4
13,6	500	1	0	2	2	2
12,2	500	1	0	2	2	2
15,8	500	1	0	2	2	0
13,6	1000	1	0	2	2	5
11,2	500	1	0	2	2	5
14,6	500	1	0	2	2	3
20,4	500	1	0	2	2	4
14,6	500	1	0	2	2	0
20	500	1	0	2	2	3
15,8	500	1	0	2	2	4
14,6	500	1	0	2	2	4
14,6	500	1	0	2	2	3
15,8	500	1	0	2	2	0
15,8	500	1	0	2	2	0
12,4	500	1	0	2	2	0
11,3	500	1	0	2	2	3
11,2	500	1	0	2	2	0
15,8	500	1	0	2	2	0
15,8	500	1	0	2	2	5
12,2	500	1	0	2	2	4
9	500	1	0	2	2	1
13,6	500	1	0	2	2	3
12,4	500	1	0	2	2	2
19	500	1	0	2	2	4
14,6	500	1	0	2	2	1
14,4	500	1	0	2	2	4
10	500	1	0	2	2	3
13,6	500	1	0	2	2	2
6	500	1	0	2	2	2
17	500	1	0	2	2	1
16	500	1	0	2	2	3
14,4	500	1	0	2	2	4
13,6	500	1	0	2	2	4

12,4	500	1	0	2	2	4
15,8	500	1	0	2	2	0
13,6	500	2	0	2	2	1
11,2	500	1	0	2	2	4
17	500	1	0	2	2	0
12,4	500	1	0	2	2	3
15,8	500	1	0	2	2	5
15,8	500	1	0	2	2	0
12,4	500	1	0	2	2	4
13,8	500	1	0	2	2	3
15,8	500	3	0	2	2	3
16	500	3	0	2	2	4
17	500	1	0	2	2	4
13,6	500	1	0	2	2	2
13,6	500	1	0	2	2	4
16	500	1	0	2	2	5
15,8	500	1	0	2	2	4
15,8	500	1	0	2	2	4
20	500	1	0	2	2	4
12,4	500	1	0	2	2	1
15,8	500	1	0	2	2	3
18	500	1	0	2	2	0
14,6	500	1	0	2	2	1
14,6	500	1	0	2	2	4
14,4	500	1	0	2	2	0
20	500	1	0	2	2	4
13,6	500	1	0	2	2	4
20	500	1	0	2	2	0
19	500	1	0	2	2	2
14	500	1	0	2	2	1
13,6	500	1	0	2	2	1
12,4	500	1	0	2	2	4
14	500	1	0	2	2	1
16	500	1	0	2	2	2
12,4	500	1	0	2	2	6
14,6	500	1	0	2	2	5
18	500	1	0	2	2	1
14,6	500	1	0	2	2	3
11,2	500	1	0	2	2	4
12,4	500	1	0	2	2	0
13,6	500	1	0	2	2	0
10	500	1	0	2	2	1
14,6	500	1	1	2	2	4
13,6	500	1	0	2	2	1
15,8	500	1	0	2	2	3
14,4	500	1	0	2	2	3

16	500	1	0	2	2	3
14,6	500	1	0	2	2	2
10	500	1	0	2	2	2
15,8	500	1	0	2	2	2
14,6	500	1	0	2	2	3
14	500	1	0	2	2	4
18	500	1	0	2	2	4
12,4	500	1	0	2	2	5
4,3	500	1	0	2	2	4
18,7	500	1	0	2	2	3
15,8	500	1	0	2	2	6
11,2	500	1	0	2	2	5
10	500	1	0	2	2	0
17	500	1	0	2	2	5
12,4	500	1	1	2	2	3
15,8	500	1	0	2	2	6
13,6	500	1	0	2	2	1
11,2	500	1	0	2	2	4
12,4	500	1	0	2	2	6
14,6	500	1	0	2	2	2
11,2	500	1	0	2	2	4
19	500	1	0	2	2	4
12,4	500	1	0	2	2	4
14,6	500	1	0	2	2	4
9	500	1	0	2	2	6
15,8	500	1	0	2	2	5
15,8	500	1	0	2	2	2
15,8	500	1	0	2	2	6
11,3	500	1	0	2	2	5
14,6	500	1	0	2	2	3
22,6	500	1	0	2	2	2
13,6	500	1	0	2	2	5
15,6	500	1	0	2	2	4
9	500	1	0	2	2	4
13,6	500	1	0	2	2	3
18	500	1	0	2	2	5
20	500	1	0	2	2	6
15,6	500	1	0	2	2	5
17	500	1	0	2	2	6
19	500	1	0	2	2	6
15	500	1	0	2	2	6
14,6	500	1	0	2	2	0
12,4	500	1	0	2	2	4
14,6	500	1	0	2	2	5
12,4	500	1	0	2	2	4
12,4	500	1	0	2	2	6

13,6	500	1	0	2	2	5
14	500	1	0	2	2	5
20	500	1	0	2	2	5
13,8	500	1	0	2	2	2
15,8	500	1	0	2	2	3
11,2	500	1	0	2	2	4
15,8	500	1	0	2	2	3
13,6	500	1	0	2	2	6
14,6	500	1	0	2	2	2
13,6	500	1	0	2	2	1
15,8	500	1	0	2	2	3
12,4	500	1	0	2	2	4
16	500	1	0	n.a	n.a	5
14,6	500	1	0	2	2	6
16	500	1	0	2	2	4
15,8	500	1	0	2	2	3
10	500	1	0	2	2	5
17	500	1	0	2	2	5
10	500	1	0	2	2	2
10	500	1	0	2	2	6
10	500	1	0	2	2	3
14	500	1	0	2	2	5
12,5	500	1	0	2	2	6
14,6	500	1	0	2	2	5
18	500	1	0	2	2	5
13,6	500	1	0	2	2	6
14,6	500	1	0	2	2	5
15,8	500	1	0	2	2	6
15,8	500	1	0	2	2	1
15,8	500	1	0	2	2	4
12,4	500	1	0	2	2	4
20	500	1	0	2	2	5
9	500	1	0	2	2	0
20	500	1	0	2	2	5
13,6	500	1	0	2	2	5
16	500	1	0	1	2	5
15,8	500	1	0	2	2	5
15,8	500	1	0	2	2	5
16	500	1	0	2	2	6
13,6	500	1	0	2	2	4
15,8	500	1	0	1	2	5
13,6	500	1	0	2	2	5
12,4	500	1	0	2	2	6
13,6	500	1	0	2	2	6
12,4	500	1	0	2	2	6
17	500	1	0	2	2	4

15,8	500	1	1	2	2	6
20	500	1	0	1	2	6
20,4	500	1	1	1	2	6

90 day mRS	Carotid Stent patent 90 day (no Rest:1/Rest: 2)	mRS most recent (score)
3	1	3
3	1	3
0	1	0
1	1	1
0	1	0
3	2	3
4	1	3
0	1	0
3	1	0
0	1	0
2	1	1
4	1	4
0	1	0
3	1	3
0	1	0
1	2	1
4	1	4
6	1	6
0	1	0
0	1	0
2	1	1
3	2	1
2	1	2
3	1	2
1	1	0
2	1	1
2	1	1
1	1	1
1	1	1
1	2	1
3	1	1
1	1	0
2	2	0
3	1	2
2	1	1

4	1	4
2	1	0
1	1	0
4	1	4
1	1	1
0	1	0
4	1	4
0	1	0
4	1	4
2	1	2
0	1	0
4	1	4
4	1	1
2	1	2
3	1	3
5	1	5
2	1	2
3	1	3
2	1	0
1	1	1
1	1	1
1	1	5
0	1	0
1	1	1
1	1	1
4	1	4
0	1	1
0	1	0
2	1	2
1	1	1
1	1	1
3	1	3
0	1	0
1	1	1
6	1	6
4	1	4
4	1	0
2	1	0
2	1	2
1	1	1
0	2	1
1	1	0
3	1	3
1	1	0
0	1	1
1	1	1

2	1	2
1	1	2
0	1	0
1	1	2
1	1	1
1	1	1
4	1	5
3	1	2
3	1	0
0	1	0
6	1	6
5	1	5
0	1	0
4	1	4
3	1	3
6	1	6
2	1	2
3	1	3
6	1	6
0	1	0
5	1	5
2	1	2
2	1	2
1	1	1
6	1	6
5	1	5
4	1	4
6	1	6
4	1	4
3	1	3
3	1	3
4	1	4
3	1	3
3	1	3
1	1	1
5	1	5
6	1	6
4	1	4
6	1	6
6	1	6
6	1	6
0	1	0
4	1	4
6	1	6
4	1	4
6	1	6

5	1	5
2	1	2
6	1	6
1	1	1
0	1	0
4	1	4
6	1	6
6	1	6
1	1	1
0	1	0
4	1	4
6	1	6
4	1	4
6	1	6
6	1	6
3	1	3
4	1	4
5	1	5
1	1	1
6	1	6
1	1	1
3	1	3
6	1	6
4	1	4
5	1	5
6	1	6
4	1	4
6	1	6
0	1	0
2	1	2
4	1	4
4	1	4
2	1	2
6	1	6
4	1	4
4	1	4
5	1	5
5	1	5
6	1	6
4	1	4
1	2	1
5	1	5
6	1	6
6	1	6
6	1	6
4	1	4

6	1	6
6	1	6
6	1	6
